# Supplementary material for: Genome-scale metabolic models highlight stage-specific differences in essential metabolic pathways in Trypanosoma cruzi
Source: PLoS Negl Trop Dis. 2020 Oct 6;14(10):e0008728. doi: 10.1371/journal.pntd.0008728 (PMC7567352; doi:10.1371/journal.pntd.0008728)
Supplement: S1 Text — (DOCX) [file pntd.0008728.s001.docx]

# **Supplementary Methods**

# **Biomass reaction for *Trypanosoma cruzi***

# The biomass reaction for *T. cruzi* was built using the similar cellular composition of *Leishmania major*, a close-related organism belonging to the same family Trypanosomatidae[[1]](https://paperpile.com/c/624NVD/7sYYX).

# Final biomass equation for *T. cruzi* iIS312 model:

# (0.4281) ala-L + (0.2557) arg-L + (0.0932) asn-L + (0.1728) asp-L + (0.0672) cys-L + (0.1458) gln-L + (0.2134) glu-L + (0.2297) gly + (0.0960) his-L + (0.1060) ile-L + (0.3271) leu-L + (0.1184) lys-L + (0.0804) met-L + (0.1049) phe-L + (0.2059) pro-L + (0.3193) ser-L + (0.2134) thr-L + (0.2550) val-L + (0.1023) gmp + (0.0593) ump + (0.0565) ergst + **(35.115) atp + (33.475) h2o** + (14.405) nadph + (1.241) 3pg + (8.066) nh4 + (0.428) g3p + (0.397) e4p + (1.236) akg + (0.445) r5p + (1.785) oaa + (2.097) accoa + (0.642) pep + (0.712) g6p__B + (2.994) pyr + (3.015) nad --> **(35.115) adp + (33.475) h + (33.475) pi** + (3.015) nadh + (2.852) co2 + (14.405) nadp + (2.097) coa

# The changes made in the biomass reaction were supported by gap-filling for some metabolites. To check the gap-filling of the biomass metabolites, we used a package in COBRApy[[2]](https://paperpile.com/c/624NVD/fVtBW) called gap-filling. This tool compares two models with respect to a specific biomass metabolite and predicts which reactions are missing to enable growth. In our case, we compared our model iIS312 with iAC560, a larger reconstruction model. After getting the list of missing reactions for each metabolite, we checked if the reactions are presented in *T. cruzi* by accessing TritrypDB[[3]](https://paperpile.com/c/624NVD/0MDhX) and Uniprot[[4]](https://paperpile.com/c/624NVD/LjNec) databases. If we could not find strong evidence for the presence of those reactions, we set the metabolite coefficient in the biomass reaction as zero. We also added to this biomass formula the metabolites of the iSR215 biomass reaction. The overlapped metabolites that had their coefficient adjusted to the values in iSR215 biomass reaction are represented in bold. The underlined metabolites represent the metabolites of iSR215 biomass reaction that were added into the iIS312 biomass formula.

# **Biomass reaction for trypomastigote model**

# As discussed in the main text, the results for deactivated genes and reactions and the fact that this stage is non-replicative suggest that nucleic acids are not important for trypomastigotes growth. For these reasons, we set up the coefficients of Pentose Phosphate Pathway (PPP) intermediate metabolites and nucleotides as zero (damp, dcmp, dgmp, dtmp, amp, cmp, gmp, ump, g3p, e4p, and r5p).

# In addition, to take into account the reduction of protein, nucleotide synthesis in the trypomastigote metabolism, we did the following changes: 1) Glutamate (glu-L): According to [[5]](https://paperpile.com/c/624NVD/yWiu), the glutamate uptake is about 5x lower in trypomastigotes than in epimastigotes. In addition, there are no findings in the literature that suggests *T. cruzi* can synthesize glutamate - glutamine is actually synthesized from this metabolite (glutamine synthetase)[[6]](https://paperpile.com/c/624NVD/7zIk). Therefore, the uptake measurements likely accurately reflect the changes of glutamate level, so we decided to lower the glutamate coefficient accordingly ; 2)Proline (pro-L): According to [[7]](https://paperpile.com/c/624NVD/Z9ct) findings, the intracellular concentration of free proline was highest in Amastigotes, followed by trypomastigotes, and epimastigotes. On the other hand, free proline transport was highest in epimastigotes, followed by trypomastigotes, and amastigotes. Since the proline intracellular concentration in trypomastigotes presents an intermediate behavior compared to amastigotes and epimastigotes, and its metabolic flux is also regulated by transporters, we decided to keep unchanged its coefficient in the trypomastigote biomass ; 3) Aspartate (asp-L): According to [[8]](https://paperpile.com/c/624NVD/pkEd), trypomastigote aspartate uptake is same as in epimastigotes ; therefore we kept unchanged its coefficient in the trypomastigote biomass ; 4) Arginine (arg-L): According to [[9]](https://paperpile.com/c/624NVD/gxyJ), T. cruzi is unable to synthesize arginine - therefore, uptake measurements are a valid way to evaluate changes in the arginine level among the stages. [[10]](https://paperpile.com/c/624NVD/G734) findings show that L-Arginine uptake in trypomastigotes were at least 30-fold lower than in epimastigotes. In addition, the same study suggests that arginine is coupled to parasite stage and replication. We decided to lower the arginine coefficient accordingly ; 5) Glutamine (gln-L): According to [[11]](https://paperpile.com/c/624NVD/H1DH), trypomastigote glutamine uptake is same as in epimastigotes ; therefore we kept unchanged its coefficient in the trypomastigote biomass.

# Finally, regarding the other amino acids and fatty acids, we decided to lower them by a factor of ½. While this may be a relatively random factor without literature support, we found that flux distribution was not extremely sensitive to the changes in coefficients as we have also tried out other factors (including ⅓ and ¼) and saw similar results.The FVA results among the reactions remained with similar intensity and same direction (see Table S7)

# The metabolites in bold had their coefficients adjusted.

(0.2141) ala-L + (0.0085) arg-L + (0.0466) asn-L + (0.1728) asp-L + (0.0336) cys-L + (0.1458) gln-L + (0.0427) glu-L + (0.1149) gly + (0.0480) his-L + (0.0530) ile-L + (0.1636) leu-L + (0.0592) lys-L + (0.0402) met-L + (0.0525) phe-L + (0.2059) pro-L + (0.1597) ser-L + (0.1067) thr-L + (0.1275) val-L + (0.0283) ergst + (35.115) atp + **(32.205) h2o** + **(13.135) nadph** + (1.241) 3pg + (8.066) nh4 + (1.236) akg + (1.785) oaa + (2.097) accoa + (0.642) pep + (0.712) g6p__B + (2.994) pyr + (3.015) nad --> (35.115) adp + **(32.205) h** + (33.475) pi + (3.015) nadh + (2.852) co2 + **(13.135) nadp** + (2.097) coa

#

# **Single reaction deletions**

# From the results (see Supplementary tables, Table S6), 42 reactions were essential for all the stage-specific models. Among them, 13 are exchange reactions and are potentially related to essential nutrients for the parasite; 18 are transport reactions and no gene-associated; and 11 are metabolic gene-associated reactions and potential drug targets for Chagas disease. Among the 11 gene-associated essential reactions, they belong to Glutamate Metabolism (1 reaction), Glycine, Serine, and Threonine Metabolism (2 reactions) and Glycolysis/Gluconeogenesis (8 reactions). In addition, 17 reactions were unique for amastigote and epimastigote, both replicative stages of *T. cruzi*. Among these reactions, 12 are gene-associated and mostly belong to Pentose Phosphate Pathway (8 reactions). Two of them belong to Purine Metabolism. Both subsystems are associated with nucleic acid synthesis. Finally, 10 essential reactions were unique for amastigote and 8 for trypomastigote. Among the unique essential reactions for amastigote, 5 reactions are gene-associated metabolic reactions. For trypomastigote, only 2 reactions are gene-associated metabolic reactions.

#

# **Double gene deletions**

# We also did double gene deletions to get the list of double essential genes for each model. The results are described in Figure SB. From the results (see Supplementary tables, Table S10 for more details). The results indicate that the amastigotes are the most susceptible stage, presenting more lethal double deletions than the other stages.

# **Comparison with experiment findings**

The prediction accuracy of iIS312 was compared against iSR215 using the experimental data used for model validation for iSR215 [[12]](https://paperpile.com/c/624NVD/qKbV), and the result is reported in Table S2. We compared the prediction accuracy of gene essentially for the iSR215 epimastigote model and iIS312 epimastigote model, and our model is shown to have an accuracy of 79%, which is the same as the prediction accuracy of iSR215. While our accuracy seems to be similar, it does not necessarily mean that our model did not improve, since the majority of the experimental data used here are from other closely related organisms such as *T. brucei* and other life cycles of *T. cruzi*, due to scarcity of the experimental data for *T. cruzi*.

#

# **References**

#

1. [Chavali AK, Whittemore JD, Eddy JA, Williams KT, Papin JA. Systems analysis of metabolism in the pathogenic trypanosomatid Leishmania major. Mol Syst Biol. 2008;4: 177.](http://paperpile.com/b/624NVD/7sYYX)

2. [Ebrahim A, Lerman JA, Palsson BO, Hyduke DR. COBRApy: COnstraints-Based Reconstruction and Analysis for Python. BMC Syst Biol. 2013;7: 74.](http://paperpile.com/b/624NVD/fVtBW)

3. [Aslett M, Aurrecoechea C, Berriman M, Brestelli J, Brunk BP, Carrington M, et al. TriTrypDB: a functional genomic resource for the Trypanosomatidae. Nucleic Acids Res. 2010;38: D457–62.](http://paperpile.com/b/624NVD/0MDhX)

4. [Consortium TU, The UniProt Consortium. The Universal Protein Resource (UniProt). Nucleic Acids Research. 2007. pp. D193–D197. doi:](http://paperpile.com/b/624NVD/LjNec)[10.1093/nar/gkl929](http://dx.doi.org/10.1093/nar/gkl929)

5. [Silber AM, Rojas RLG, Urias U, Colli W, Alves MJM. Biochemical characterization of the glutamate transport in Trypanosoma cruzi. Int J Parasitol. 2006;36: 157–163.](http://paperpile.com/b/624NVD/yWiu)

6. [Crispim M, Damasceno FS, Hernández A, Barisón MJ, Pretto Sauter I, Souza Pavani R, et al. The glutamine synthetase of Trypanosoma cruzi is required for its resistance to ammonium accumulation and evasion of the parasitophorous vacuole during host-cell infection. PLoS Negl Trop Dis. 2018;12: e0006170.](http://paperpile.com/b/624NVD/7zIk)

7. [Tonelli RR, Silber AM, Almeida-de-Faria M, Hirata IY, Colli W, Alves MJM. L-proline is essential for the intracellular differentiation of Trypanosoma cruzi. Cell Microbiol. 2004;6: 733–741.](http://paperpile.com/b/624NVD/Z9ct)

8. [Canepa GE, Silber AM, Bouvier LA, Pereira CA. Biochemical characterization of a low-affinity arginine permease from the parasite Trypanosoma cruzi. FEMS Microbiol Lett. 2004;236: 79–84.](http://paperpile.com/b/624NVD/pkEd)

9. [Silber AM, Colli W, Ulrich H, Alves MJM, Pereira CA. Amino acid metabolic routes in Trypanosoma cruzi: possible therapeutic targets against Chagas’ disease. Curr Drug Targets Infect Disord. 2005;5: 53–64.](http://paperpile.com/b/624NVD/gxyJ)

10. [Pereira CA, Alonso GD, Ivaldi S, Silber A, Alves MJM, Bouvier LA, et al. Arginine metabolism in Trypanosoma cruzi is coupled to parasite stage and replication. FEBS Lett. 2002;526: 111–114.](http://paperpile.com/b/624NVD/G734)

11. [Damasceno FS, Barisón MJ, Crispim M, Souza ROO, Marchese L, Silber AM. L-Glutamine uptake is developmentally regulated and is involved in metacyclogenesis in Trypanosoma cruzi. Mol Biochem Parasitol. 2018;224: 17–25.](http://paperpile.com/b/624NVD/H1DH)

12. [Roberts SB, Robichaux JL, Chavali AK, Manque PA, Lee V, Lara AM, et al. Proteomic and network analysis characterize stage-specific metabolism in Trypanosoma cruzi. BMC Syst Biol. 2009;3: 52.](http://paperpile.com/b/624NVD/qKbV)
