# Supplementary figures and images for: Genome-scale metabolic models highlight stage-specific differences in essential metabolic pathways in Trypanosoma cruzi
Source: PLoS Negl Trop Dis. 2020 Oct 6;14(10):e0008728. doi: 10.1371/journal.pntd.0008728 (PMC7567352; doi:10.1371/journal.pntd.0008728)

# Trypanosoma cruzi

## Metabolic Network

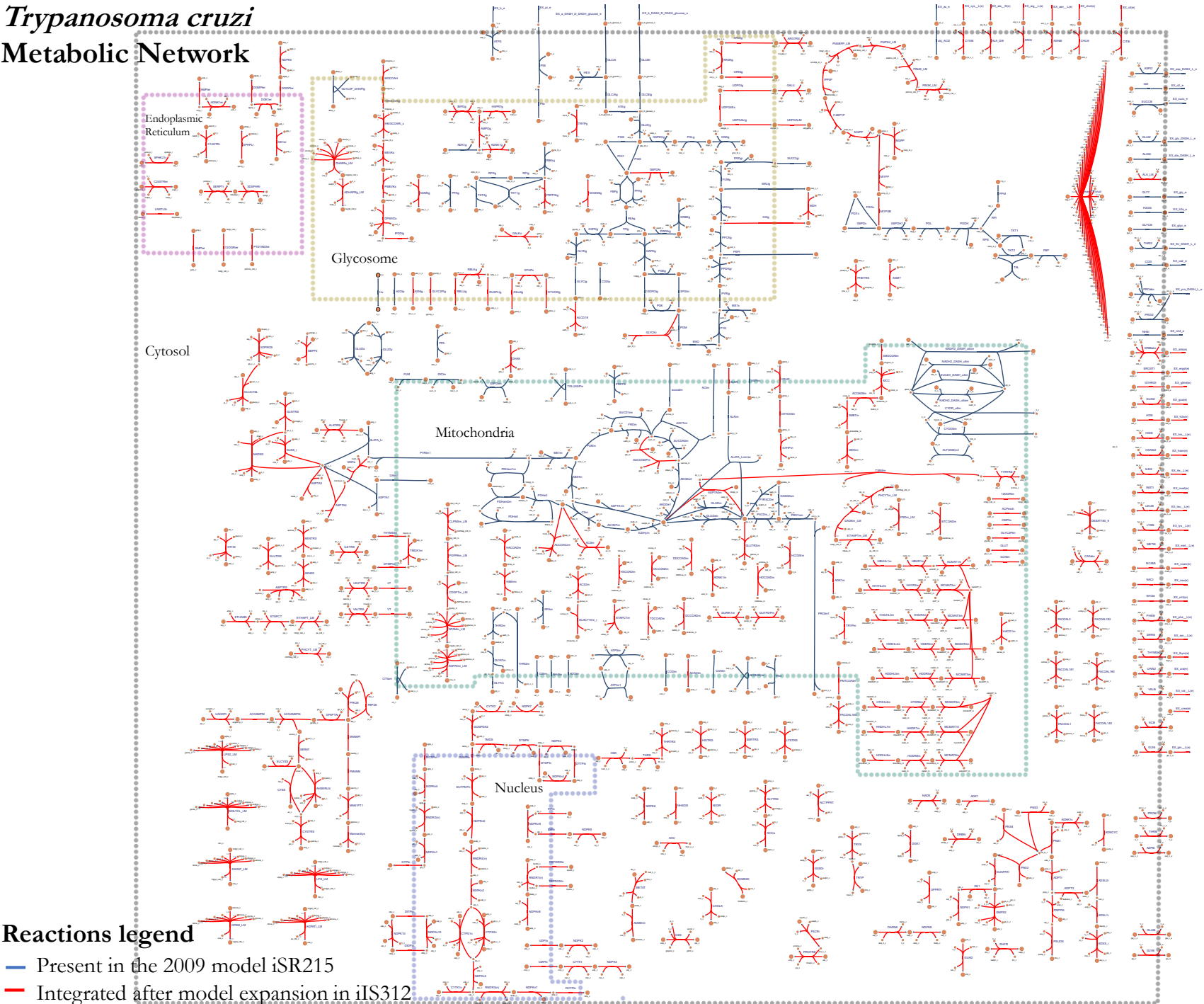

Supplement: S1 Fig — (PDF) [file pntd.0008728.s016.pdf]

# FBA results - Espimastigote

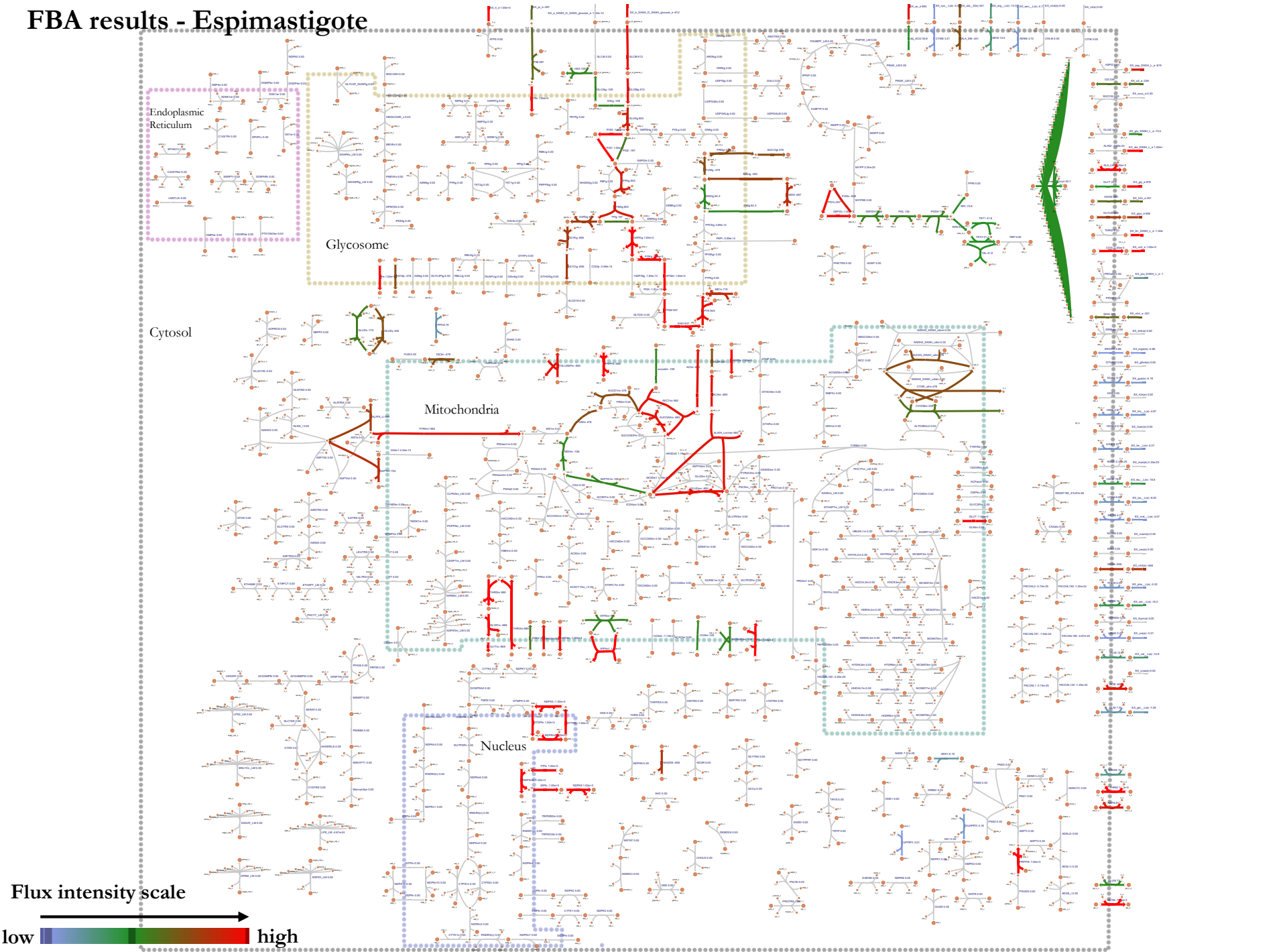

Supplement: S2 Fig — (PDF) [file pntd.0008728.s017.pdf]

# FBA results - Amastigote

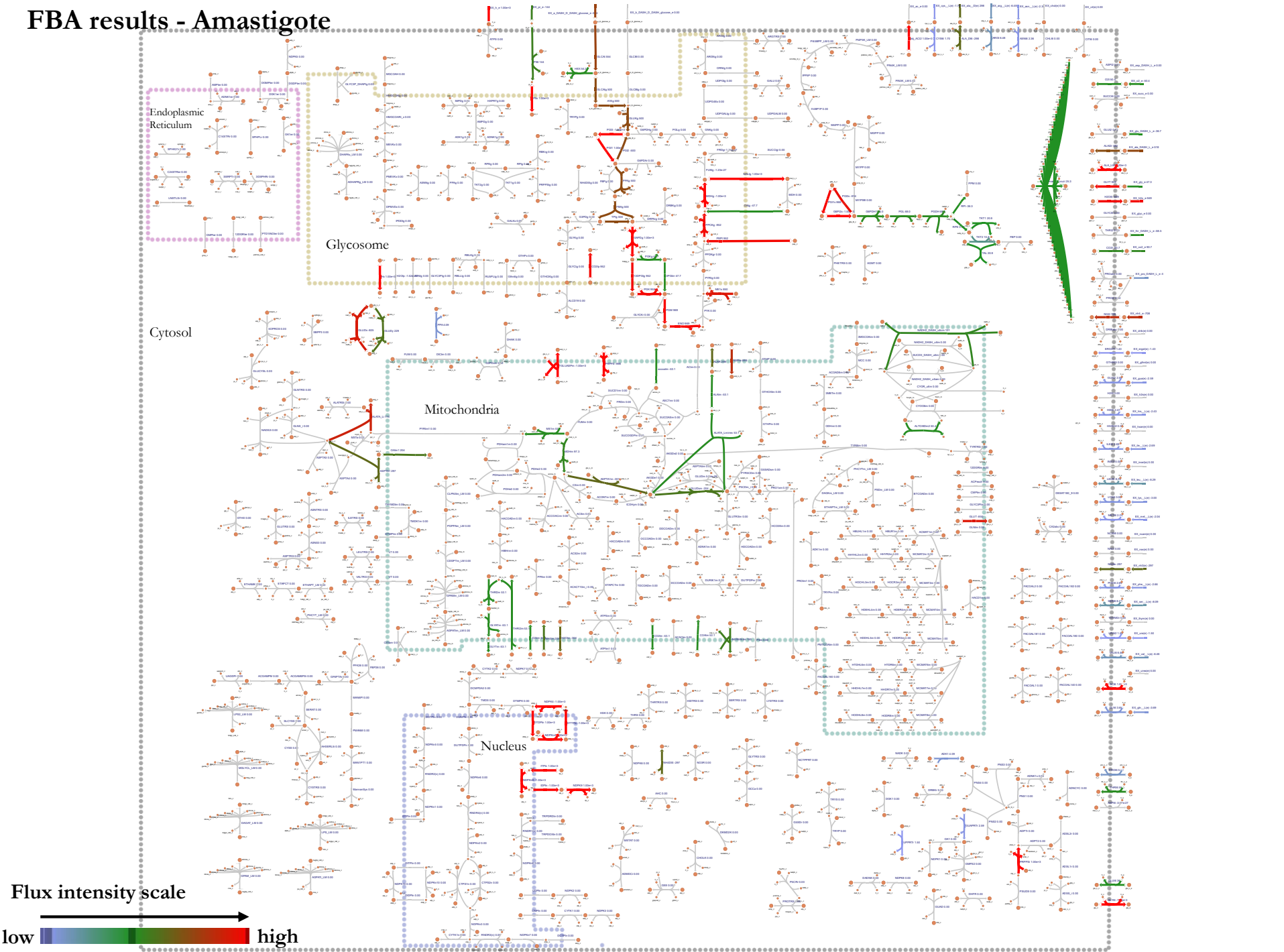

Supplement: S3 Fig — (PDF) [file pntd.0008728.s018.pdf]

# FBA results - Trypomastigote

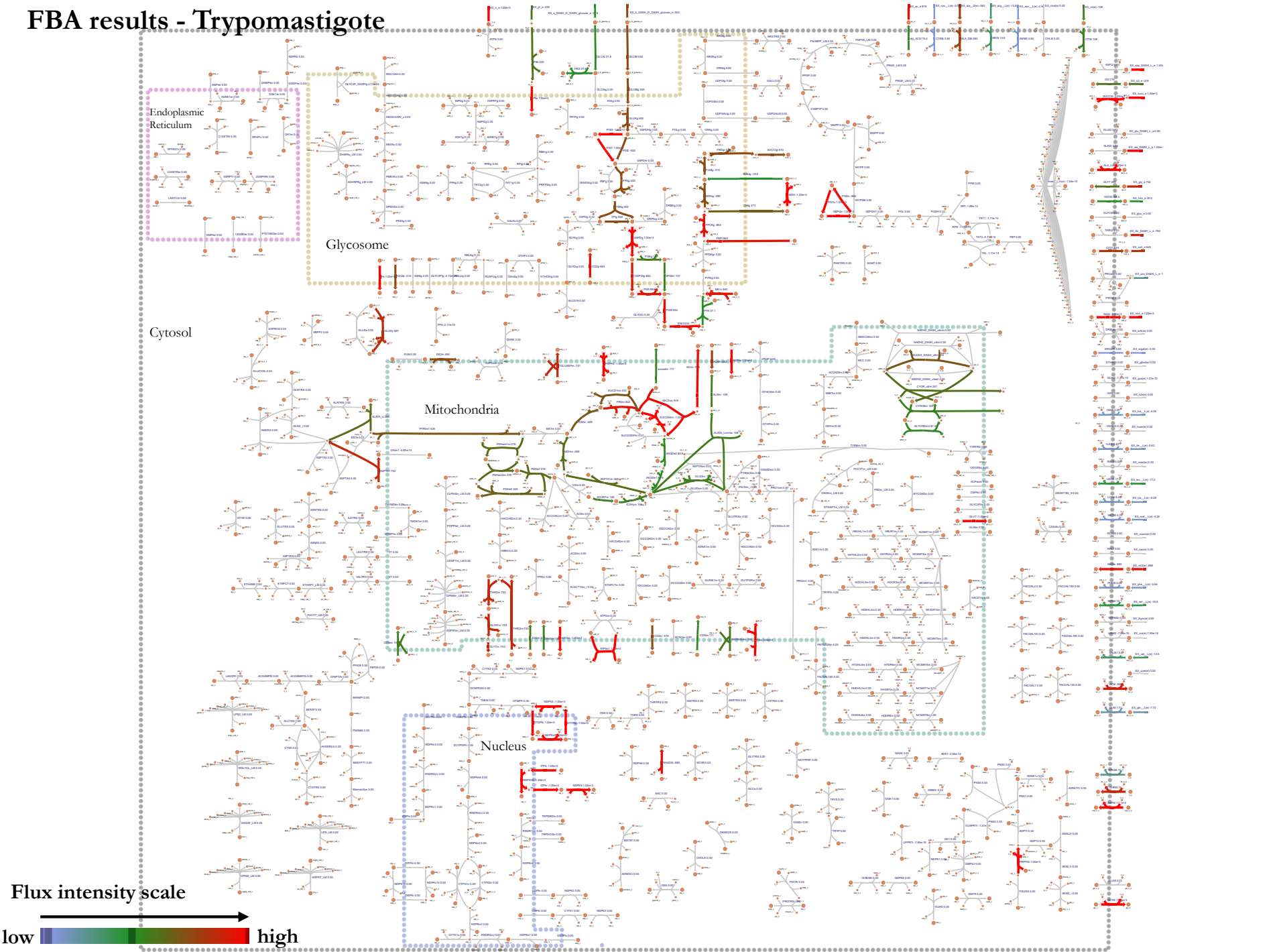

Supplement: S4 Fig — (PDF) [file pntd.0008728.s019.pdf]

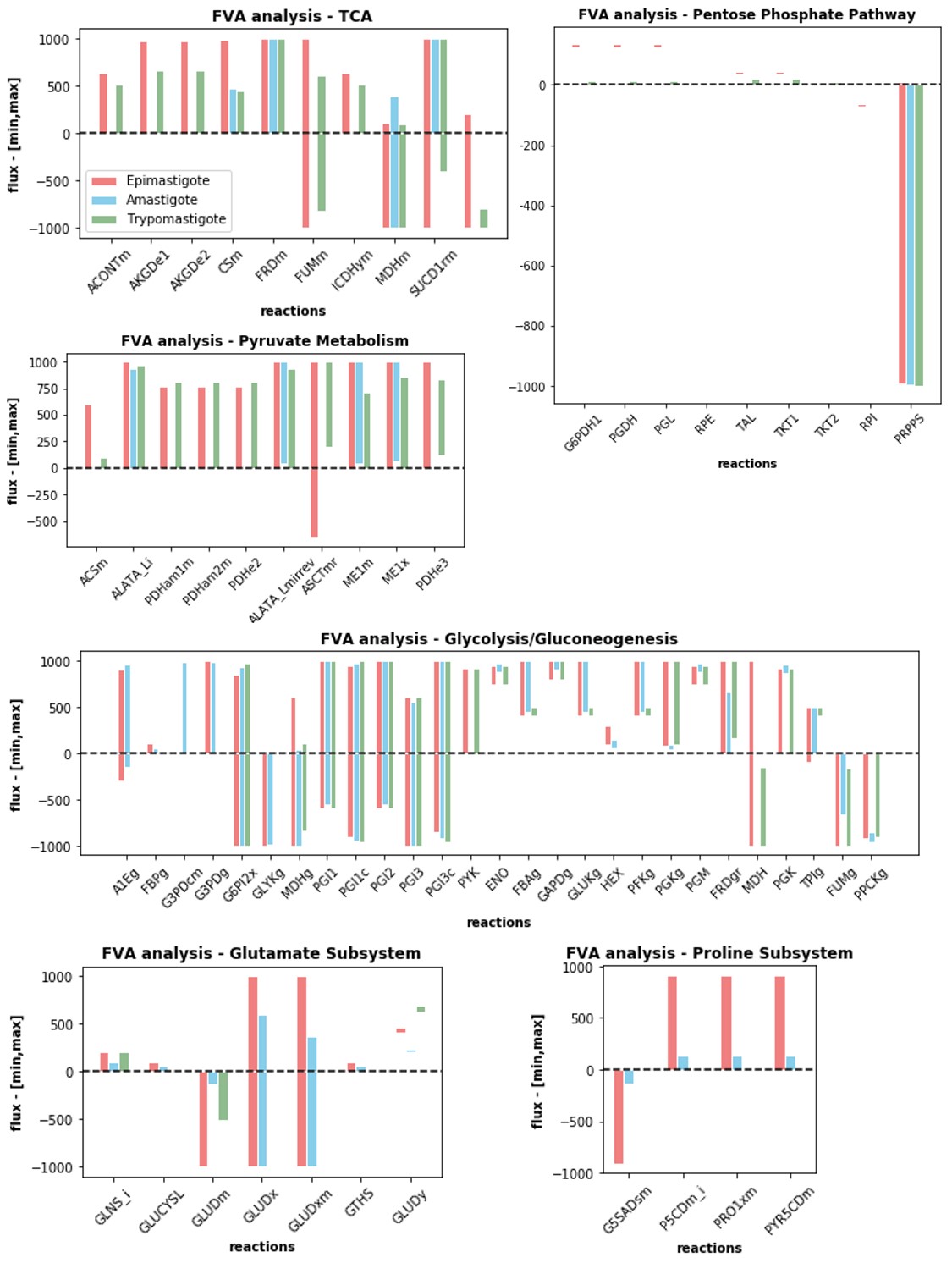

Supplement: S5 Fig — (JPG) [file pntd.0008728.s020.jpg]
