# Supplementary material for: Genome-scale metabolic models highlight stage-specific differences in essential metabolic pathways in Trypanosoma cruzi
Source: PLoS Negl Trop Dis. 2020 Oct 6;14(10):e0008728. doi: 10.1371/journal.pntd.0008728 (PMC7567352; doi:10.1371/journal.pntd.0008728)
Supplement: Attachment — Submitted filename: Rebutal letter_JLSN.docx [file pntd.0008728.s021.docx]

**Responses to reviewers comments and suggestions**

Reviewer #1: The manuscript by Shiratsubaki et al. describes the reconstruction of an updated genome scale metabolic model of Trypanosoma cruzi, iIS3142. The authors used the expansion of the genome annotation of *T. cruzi* to extend a previous model of core metabolism iSR215, while using also information from the *Leishmania* model iAC560. Using transcriptome data for the different lifecycle stages of *T. cruzi*, they made lifestage specific models. This allowed the authors to compare the metabolic networks in the lifestages and how metabolism sustains growth. They validated some of the network differences by measuring enzyme activities in the different lifestages.

This is a very relevant study as this extends the knowledge on the metabolic capabilities of *T. cruzi*. Metabolic enzymes could be potential drug targets and models can help in identifying the most promising drug targets in metabolism.

While it is an interesting paper, there are a few major points that I would like to see addressed – also to make it more accessible to a non-GEM/FBA readership

Major points

1. Although there is an intrinsic value in extending the previous model, it is not clear to me whether the new reconstruction improved predictions or not. The authors should make a comparison of the prediction capabilities between both models and comment why the new model is better or worse. For example, for the genes which are common to both models, how many of them are accurately predicted to be essential using both models.

We mentioned the prediction accuracy of essential genes in the supplementary text and table S4. It was originally between the iSR215 and iIS312 pan model, but as the reviewer has pointed out, it makes more sense to be looking at stage-specific models instead of pan models. Hence, we have now updated the comparison to be between the epimastigote models of iSR215 and iIS312 using the experimental data used for validation in the iSR215 paper [[1]](https://paperpile.com/c/81Zq7k/def2n). iSR215 achieved an accuracy of 79% and our epimastigote model also achieved an accuracy of 79%. However, this does not necessarily mean that our model did not improve, because the majority of the experimental data used for comparison from the iSR215 paper was from related organisms such as *T. brucei*, or other life stages of *T. cruzi*, due to the scarcity of experimental data for *T. cruzi*. We reported the comparison results in the supplementary text (Supplementary Table S4).

2. I wonder why the authors considered the biomass objective as the function to optimize for all the stages. This make sense for epimastigotes and amastigotes that replicate. However, it does not make sense for trypomastigotes which do not replicate. Non-replicating cells need to produce considerably less compounds, mainly for maintenance and pathogenicity and it seems very unlikely that the cell is trying to optimize biomass yield as they don’t replicate. Although the authors modified the biomass equations as stated in the supplementary material methods, it seems that this change is insufficient. For example, the new biomass equation still considers the same coefficients for amino acids. It is highly unlikely that replicating and non-replicating cell need the same amount of amino acids for protein synthesis. Please comment on this.

We agree with the reviewer that special considerations in the trypomastigote stage biomass reaction should account for its non-replicative characteristic. We have updated the model modifying the coefficients of metabolites where we could find literature evidence. Due to the scarcity of available data, we were only able to find changes in uptake rates of certain amino acids in the trypomastigote stage. While this may not be an accurate reflection of the changes in biomass, this could be an indirect measurement of the extent of changes of certain metabolites. We made the following changes based on the data available:

**Glutamate:** According to [[2]](https://paperpile.com/c/81Zq7k/kpegT), uptake is about 5x lower in trypomastigotes than in epimastigotes. In addition, there are no findings in the literature that suggests *T. cruzi* can synthesize glutamate - glutamine is actually synthesized from this metabolite (glutamine synthetase). Therefore, the uptake measurements likely accurately reflect the changes of glutamate level, so we decided to lower the glutamate coefficient accordingly.

**Proline**: According to [[3]](https://paperpile.com/c/81Zq7k/7F5O8) findings, the intracellular concentration of free proline was highest in Amastigotes (8.09 ± 1.46 mM), followed by trypomastigotes (3.81 ± 1.55), and intracellular epimastigote‐like forms (0.45 ± 0.06 mM). On the other hand, free proline transport was highest in epimastigotes, followed by trypomastigotes, and amastigotes. Since the proline intracellular concentration in trypomastigotes presents an intermediate behavior compared to amastigotes and epimastigotes, and its metabolic flux is also regulated by transporters, we decided to keep unchanged its coefficient in the trypomastigote biomass.

**Aspartate:** According to [[4]](https://paperpile.com/c/81Zq7k/gGlRb), trypomastigote aspartate uptake is the same as in epimastigotes ; therefore we kept unchanged its coefficient in the trypomastigote biomass.

**Arginine**: According to [[5]](https://paperpile.com/c/81Zq7k/bk1x5), *T. cruzi* is unable to synthesize arginine - therefore, uptake measurements are a valid way to evaluate changes in the arginine level among the stages. [[6]](https://paperpile.com/c/81Zq7k/ZeOqC) findings show that L-Arginine uptake in trypomastigotes were at least 30-fold lower than in epimastigotes. In addition, [[6]](https://paperpile.com/c/81Zq7k/ZeOqC) also suggests that arginine is coupled to parasite stage and replication. We decided to lower the arginine coefficient accordingly.

**Glutamine**: According to [[7]](https://paperpile.com/c/81Zq7k/NjKc4), trypomastigote glutamine uptake is the same as in epimastigotes ; therefore we kept unchanged its coefficient in the trypomastigote biomass.

Regarding the other metabolites, we decided to lower them by a factor of ½ to take into account the reduction of protein, nucleotide and fatty acid synthesis in this non-replicative stage. While this may be a relatively arbitrary factor without literature support, we found that flux distribution was not extremely sensitive to the changes in coefficients as we have also tried out other factors (including ⅓ and ¼) and saw similar results. The FVA results among the reactions remained with similar intensity and same direction (please check the new results in Table S8).

New biomass reaction:

(~~0.4281~~ 0.2141) ala-L + (~~0.2557~~ **0.0085**) **arg-L** + (~~0.0932~~ 0.0466) asn-L + (0.1728) **asp-L** + (~~0.0672~~ 0.0336) cys-L + (0.1458) **gln-L** + (~~0.2134~~ **0.0427**) **glu-L** + (~~0.2297~~ 0.1149) gly + (~~0.0960~~ 0.0480) his-L + (~~0.1060~~ 0.0530) ile-L + (~~0.3271~~ 0.1636) leu-L + (~~0.1184~~ 0.0592) lys-L + (~~0.0804~~ 0.0402) met-L + (~~0.1049~~ 0.0525) phe-L + (0.2059) **pro-L** + (~~0.3193~~ 0.1597) ser-L + (~~0.2134~~ 0.1067) thr-L + (~~0.2550~~ 0.1275) val-L + (~~0.0565~~ 0.0283) ergst + (35.115) atp + (32.205) h2o + (13.135) nadph + (1.241) 3pg + (8.066) nh4 + (1.236) akg + (1.785) oaa + (2.097) accoa + (0.642) pep + (0.712) g6p__B + (2.994) pyr + (3.015) nad --> (35.115) adp + (32.205) h + (33.475) pi + (3.015) nadh + (2.852) co2 + (13.135) nadp + (2.097) coa

Therefore, we reported the trypomastigote biomass changes in the Supplementary Methods text from line 36 to 62.

3. Despite that the model was validated regarding by-products and reaction essentiality, the model should also be validated in other aspects too to ensure a basic functioning and usability. Recently, Memote, a suite to assist in the assessment of genome-scale models, was published. We strongly suggest the authors to use this suite to further validate the model and include the results in the manuscript (see: <https://doi.org/10.1038/s41587-020-0446-y>)

We have assessed iIS312 via MEMOTE and reported some of the key statistics in the main text (lines 428-434):

“We have also evaluated the iIS312 model via a model testing suit MEMOTE [[8]](https://paperpile.com/c/81Zq7k/56jQr). MEMOTE reported that the stoichiometric consistency, metabolite connectivity, mass and charge balance and metabolite and reaction identifier namespace to be >95%. The annotation information and sources are very specific for our model (as *T. cruzi* has its own database), therefore cannot be properly evaluated by MEMOTE. However, all annotation information of our model is available in Table S1. Despite the annotation score, MEMOTE still reported an overall score of 81% for iIS312.”

4. It is not clear to me how big is the non-metabolic effect regarding inactivation of genes. I would like to have a bigger picture regarding transcriptomics and the genome-scale model. For example, how many genes overlap between the transcriptomics and the genome-scale model? How many genes are present in the transcriptomics and missing in the model? How many genes were deactivated according to the transcriptomics but they cannot be taken into account because they are not in the model? Do the authors expect only metabolic (and not regulatory) changes among the different stages?

Out of all 11,347 genes in the transcriptomics data (2,221 have E.C. number), and only 312 are included in our model. The reason that only a small portion of genes are included in the model is that many genes with E.C. numbers either had a broad description E.C. or we could not find more supporting findings in the literature about their role and specific reaction in *T. cruzi* metabolism. Regarding the deactivated genes - take the amastigote stage as an example, 1,625 out of 11,347 genes (total number of genes in the transcriptomics) should be deactivated according to our methodology, while this ratio is 467 out of 2,221 for the genes with E.C. numbers; in addition - still for the amastigote stage, the majority of the deactivated genes are well covered in our model: 68 genes should be deactivated according to our threshold, but only 59 deactivated genes are present in our model. As we note in the last paragraph of the discussion, one of the limitations is the lack of annotation in *T. cruzi* and we expect significant expansion of the model as more metabolic genes are discovered in the future. We believe regulatory changes definitely exist across stages, but our model is only focused on metabolic genes as we have also mentioned in the discussion. To make these clearer for the readers, we have now expanded a bit more on these limitations in the discussion (lines 402-407):

“It is important to note that the current GEM only delineates the metabolic capability of *T. cruzi,* without taking into account many other functions such as regulation, gene expression or infection. But this model will be a good starting point for building more comprehensive models such as a metabolic and gene expression model [34] and additional knowledge including structural information of metabolic enzymes [35] and regulatory functions can also be incorporated in the future.”

In addition, the Table S5 was edited and includes now a column B that informs if the gene is added or not in the iIS312 model.

5. The authors at several points in the manuscript state that metabolism could yield potential drug targets. While I agree with this, many metabolic pathways and enzymes are also operational in the human host. Therefore, the authors should discuss how side-effects could be prevented (e.g. because some pathways or enzymes may be less important in host metabolism)(see e.g. <https://doi.org/10.1038/msb.2010.115> and <https://doi.org/10.1038/srep40406>)

We thank the reviewer for the valuable input. We have added a couple of sentence discussing the need to prevent off-target and adverse effects on host when the parasite metabolism is the target (lines 349-352):

“To select potential drug targets for further experimental validation, it is also important to minimize collateral damage to host cells, by focusing on enzymes that do not have homologs in the host [[9]](https://paperpile.com/c/81Zq7k/7hTE2), or enzymes that are essential to the pathogen but not for the host [[10]](https://paperpile.com/c/81Zq7k/NHDMp).”

6. It seems that the authors calculated the results of Fig 2C using parsimonious FBA. This is not fully correct to explore the potential of a metabolic network regarding products. Flux variability analysis should be performed to explore which metabolic products can be produced at optimal state. Also, it would be interesting to see what products must be produced at optimal state. For a reference regarding studying the potential for metabolic products, see <https://doi.org/10.1038/nbt.3703>

We have performed the FVA analysis for stage-specific models- the detailed results can be found in Table S8. The metabolite is considered secreted if the maximum possible flux through the fermentation product exchange reaction was positive according to the previous study [[11]](https://paperpile.com/c/81Zq7k/UKMEw). The updated results are summarized in the figure 2C and Table S3 now. We have updated the results in the main text and the method section accordingly.

Main text (lines 135-147):

“The potential of end products secretion was predicted by the iIS312 stage-specific models through flux variability analysis (FVA [[12]](https://paperpile.com/c/81Zq7k/MM2AP)), a method that is used to investigate the ranges of reaction fluxes for alternative optimal solutions. The prediction of end products fit the expected spectrum of metabolite secretion (Fig. 2C and Table S3), supporting the validity of the models (see Methods). We also compared the experimental observations with the predicted metabolite secretion by stage-specific models (details of stage-specific models discussed in the next section) whenever the literature evidence is available (Fig. 2c). No literature evidence was available on glycine and CO_2_ for *T. cruzi,* we included them as they were found in a closely related organism *T. brucei* [*[13]*](https://paperpile.com/c/81Zq7k/HHUku). Although citrate showed up for the amastigote and epimastigote models, it was not included in Figure 2c due to lack of experimental data on it. The stage-comparison suggests that overall consistency is relatively high, with some minor inconsistencies, which could be potentially due to knowledge gaps and differences in experimental and simulated media.”

Method (lines 522-525):

“The end products were generated by FVA in COBRApy [[14]](https://paperpile.com/c/81Zq7k/7Xdk). The biomass reaction was set up as the objective function. The metabolite is considered secreted if the maximum possible flux through the fermentation product exchange reaction was positive according to a previous study [[11]](https://paperpile.com/c/81Zq7k/UKMEw). The secretion profile for this analysis is shown as presence/absence on Table S3.”

7. Why is the Pan-model part of Fig 2C? This model is not biologically relevant as this is not a lifecycle stage that exists. It could, theoretically, be interesting to show the full metabolic potential of T. cruzi. But a flux analysis is a functional analysis and should be restricted to the actual lifestages. This comment is also relevant for the analysis of PAN for deletions.

In addition, it would be more informative if Fig 2C could compare the FBA outcome per lifestage to what is known of the excreted products for the different lifestages from literature.

We agree with review that the predictions should be performed using models representing actual life stages. We have now updated Figure 2C and Table S3 and removed the simulation results for pan-models. We were able to find some limited information on end products secretion across stages from references 16-20 in the manuscript, and we added this information to figure 2C to allow stage-specific comparison. However, as shown in the figure, information was only available for certain stages for many metabolites due to the scarcity of data for *T. cruzi*, and sometimes we also had to include data from closely related organisms such as *T. brucei* (such as CO2 and glycine). For other functional analysis including essential gene analysis and FVA analysis, the pan model was not used as shown in Table S8-S11, and has been removed from table S4.

Minor points

a. Line 119 and Fig 2a: Please explain why an extracellular compartment is part of a GEM of a cell. This may be logic to modellers, but a short explanation would make the paper wider accessible.

We have added a sentence explaining why extracellular compartments are needed (lines 123-125):

“The extracellular compartment is included in order to simulate nutrient uptake through transport reactions from extracellular space.”

b. The manuscript often refers to metabolic BYproducts. This suggests that there a more relevant purpose of metabolism (making growth possible!), but the authors never make this explicit in the manuscript

We agree with the reviewer´s observation, and in fact, “byproduct” might not be the best word since it suggests that these metabolites could be used for other purposes that those established as the objective function. Therefore, for the end products of the metabolism we replaced “byproducts” for “metabolic end products”.

c. At line 128 (now 88), FBA is first mentioned. While the methods section explains it further and provide useful references, for the less-informed reader we would like to see a short overall explanation of what is the purpose of this method and what type of questions this approach will answer. This should include the objective function that is used (a biomass equation) and the quasi-steady state assumption. The authors can keep it short, but it should convey the essence of the approach.

We have added a short explanation of the FBA method in the introduction (lines 88-81):

“Flux balance analysis (FBA), one of the most commonly used methods in GEM analysis, is used in this study to investigate the GEMs built. FBA is able to calculate the flux distributions that optimize the objective function (such as biomass function) with the given constraints, including steady-state assumption, network topology and nutrient uptake rates [[15]](https://paperpile.com/c/81Zq7k/tB6iZ).”

d. The legend to Fig 2C should be extended with explain what the PAN model is (if it stays in Fig 2C) and why these excreted metabolism are listed in the model (previous experimental data?)

We have removed the pan model from Figure 2C and added stage-specific comparison on the excreted end products. The excreted metabolites were listed in the figure because evidence was available in the literature about experimental data in *T. cruzi* and *T. brucei,* and the transporter reactions are also present in our model. According to these data, succinate and acetate were excreted by all the stages [[16]](https://paperpile.com/c/81Zq7k/K2Wxx), L-alanine was found as an end product of epimastigote forms but not for amastigote forms [[17]](https://paperpile.com/c/81Zq7k/67S5Q), glycerol was found as an end product for amastigotes but not for epimastigotes [[16]](https://paperpile.com/c/81Zq7k/K2Wxx), CO2 and glycine were secreted by *T. brucei* (closely related organism), and NH3 was found in epimastigotes secretion [[18]](https://paperpile.com/c/81Zq7k/TyBDo).

e. Fig 3: There are no reactions that are deactivated in all lifestages. Was this expected?

The methodology MEM applied for the transcriptomics integration and construction of stage-specific models (described in the main text in lines 484-519) defines a threshold (line 492) as the 25th percentile of the overall gene expression value distribution. Because the threshold is conservative, we did not have any case where a gene was not expressed for all life stages (please, see Table S5). If the threshold were more aggressive (e.g. 90th percentile), we would see cases of gene not expressed for all the life stages. Therefore, this is expected for choosing a conservative threshold.

f. Fig 4: It would be helpful to include substrates, end-products and directions of flux. In the amastigotes, there is no substantial flux from pyruvate to TCA, so the substrate for lower part are the amino acids, but this does not become immediately clear from the figure. Especially as line 269 (now 298) mentions a gluconeogenic flux.

As suggested by the reviewer, we have added the metabolite names and directions of flux and hopefully that can help with interpretation of the figure. The gluconeogenic flux mentioned in line 298 is actually an experimental finding - it is not currently consistent with our flux prediction due to the fact that we had to add hexokinase back into the model to sustain growth during the gap-filling process (see detailed response in comment h). But in the future if this aspect of hexokinase could be resolved we expect to see consistency between experimental finding and flux prediction regarding the gluconeogenic flux.

g. Fig 5: Please make the order of the lifecycle stages the same as Fig 4 for easy reference.

The order of stages has been fixed for Figure 5.

h. Fig 5: How is hexokinase a validation of the models? It has a high flux intensity in all models according to figure 4.

Hexokinase (HK) was actually first deactivated in the amastigote model based on the transcriptomics data, which is consistent with the experimental data shown in Fig 5. But HK was added back to the amastigote model in the process of gap filling as growth was disabled after HK deletion, so the HK flux shown in Figure 4 is not an accurate reflection of the real flux distribution. We believe this could potentially be due to unknown pathways that can enable glycolysis without HK (such as transporters that directly uptake glycolysis intermediate from the environment), and If such pathways can be added to the model, we will be able to delete HK without disabling growth, and observe the same trend between flux and experimental data for HK. We apologize for the confusion, and have added a much more detailed explanation in the text (288-293):

“HK should have been deactivated in amastigote based on transcriptomics data, but was added back during the gap-filling process as its deletion disabled growth, possibly due to the unknown alternative pathways that are not present in the model. We expect to see consistency between HK flux prediction and measurements if such alternative pathways can be discovered and added to the model in the future.”

i. Line 391: the medium that was used for growth simulation was based on earlier in silico studies? Why didn’t the authors use the composition of the medium that was used in the validation experiments.

While we’d like to keep simulation and actual media consistent, there are certain constraints in doing that - the actual media used in involved complex media that contain materials of biological origin (liver infusion tryptose medium, fetal bovine serum) instead of defined media, which makes simulation challenging. We instead opened all exchange reactions and allowed the model to intake any nutrients needed. While this simulation still provides valuable insights into stage-specific differences, we agree that this may not be the most accurate reflection of the stage-specific metabolism under the experimental conditions presented, and we have already included in the discussion on this limitation (lines 396-401):

“Another limitation of this work is that the transcriptomics data was collected from different complex growth media for different stages, yet we used the same simulation medium across stages, due to the lack of defined growth media for *T. cruzi*. This could potentially be addressed in the future with the development of defined media and corresponding stage-specific transcriptomics data to ensure consistency between simulation and actual growth.”

j. Line 421: Does MEM include gap-filling? Or gap-filling was performed after MEM?

Gap filling was performed twice for different purposes. It was first performed during the reconstruction process when we were building the pan model iIS312 and its biomass from iSR215 (*T. cruzi*, 2009) and iAC560 (*L. major*, 2008). Gap-filling was performed again to generate strains-specific models after MEM (if their growth were null). The stage-specific models were gap filled by using the pan model and iAC560. The gap-filling process is described in lines 289 and 517 in the main text, and the section “Biomass reaction of *T. cruzi*” in the Supplementary Methods text.

k. Lines 447-456: The authors say that some essential genes were deactivated in the model due to low gene-expression levels. Therefore, they removed a few reactions which made the model infeasible. To restore growth, they then do gap-filling using the template model. Why they didn’t used the BiGG database for the gap-filling procedure? In this way they can expand the search for alternative pathways as they suggest

As mentioned in item j, the gap filling was performed twice: 1) while we were building the pans model iIS312, and 2) after transcriptomics integration to build stage specific models in which the growth was null after gene deactivation.

For 1), we used the model iAC560 (560 genes and 1,112 reactions) - *Leishmania major* [[19]](https://paperpile.com/c/81Zq7k/4keyf), closely related protozoan that belongs to the same family Trypanosomatidae - as the template model to perform gap-filing. We redid this step by using BiGG universal model instead, as suggested by the reviewer, but did not discover new alternative pathways

For 2), we use the iAC560 and iIS312 pan model as a template for the gap filling. We tried BiGG universal model instead to check if the results would be different, but did not discover new alternative pathways.

Reviewer #2: In this manuscript, authors a genome scale metabolic model with focus on targets for development of drugs against Chagas disease, a neglected disease, in which currently two drugs are used -Nifurtimox nd Benznidazol- with several side/undesirable effects. They take into account the expression in the different stages, extracted from previously published transcriptomic studies. Particularly, they describe metabolic differences among stages, some of them already validated in previous publications, and by measuring the activity of the enzymes G-6-P dehydrogenase, malic enzyme, hexokinase, glutamate dehydrogenase and citrate synthase, confirming their predicted results.

I consider that this manuscript gives very relevant information about metabolic fluxes in the relevant stages in mammals (amastigotes and trypomastigotes), which gives important clues about valid drug targets in T. cruzi. In my opinion this paper should be accepted after minor changes:

- Figure 1, "epimastigote" (instead of "espimastigote")

We have fixed this issue in Figure 1.

- Line: 191: "amastigotes" (instead of "amastigote")

We have fixed this issue in the text.

- authors should consider to discuss about the role of malic enzymes in amastigotes, i.e.: anaplerotic reaction vs. NADPH source, taking into acount that PPP is not so active at this stage.

We appreciate the reviewer’s feedback and have added into the manuscript a paragraph to further discuss the role of malic enzymes in amastigotes (380-387):

“We observed that the malic enzyme (ME) seems to play an important role in the amastigotes metabolism. ME is a NADPH dependent enzyme and was already found more expressed in amastigotes forms [[20]](https://paperpile.com/c/81Zq7k/678uU). NADPH is important to support anabolic reactions such as the *de novo* synthesis of fatty acids [[21]](https://paperpile.com/c/81Zq7k/QkdN1), important for the building of glycosylphosphatidylinositol (GPI) molecules [[22]](https://paperpile.com/c/81Zq7k/VkLMt). Due to the lower activity of G6PDH linked to the absence of glycolytic metabolism in these forms, the ME in amastigotes probably plays an important role in the supply of NADPH for the parasite.”

1.	[Roberts SB, Robichaux JL, Chavali AK, Manque PA, Lee V, Lara AM, et al. Proteomic and network analysis characterize stage-specific metabolism in Trypanosoma cruzi. BMC Syst Biol. 2009;3: 52.](http://paperpile.com/b/81Zq7k/def2n)

2.	[Silber AM, Rojas RLG, Urias U, Colli W, Alves MJM. Biochemical characterization of the glutamate transport in Trypanosoma cruzi. Int J Parasitol. 2006;36: 157–163.](http://paperpile.com/b/81Zq7k/kpegT)

3.	[Tonelli RR, Silber AM, Almeida-de-Faria M, Hirata IY, Colli W, Alves MJM. L-proline is essential for the intracellular differentiation of Trypanosoma cruzi. Cell Microbiol. 2004;6: 733–741.](http://paperpile.com/b/81Zq7k/7F5O8)

4.	[Canepa GE, Silber AM, Bouvier LA, Pereira CA. Biochemical characterization of a low-affinity arginine permease from the parasite Trypanosoma cruzi. FEMS Microbiol Lett. 2004;236: 79–84.](http://paperpile.com/b/81Zq7k/gGlRb)

5.	[Silber AM, Colli W, Ulrich H, Manso Alves MJ, Pereira CA. Amino Acid Metabolic Routes in Trypanosoma cruzi: Possible Therapeutic Targets Against Chagas; Disease. Current Drug Targets - Infectious Disorders. 2005;5: 53–64.](http://paperpile.com/b/81Zq7k/bk1x5)

6.	[Pereira CA, Alonso GD, Ivaldi S, Silber A, Alves MJM, Bouvier LA, et al. Arginine metabolism in Trypanosoma cruzi is coupled to parasite stage and replication. FEBS Lett. 2002;526: 111–114.](http://paperpile.com/b/81Zq7k/ZeOqC)

7.	[Damasceno FS, Barisón MJ, Crispim M, Souza ROO, Marchese L, Silber AM. L-Glutamine uptake is developmentally regulated and is involved in metacyclogenesis in Trypanosoma cruzi. Mol Biochem Parasitol. 2018;224: 17–25.](http://paperpile.com/b/81Zq7k/NjKc4)

8.	[Lieven C, Beber ME, Olivier BG, Bergmann FT, Ataman M, Babaei P, et al. MEMOTE for standardized genome-scale metabolic model testing. Nat Biotechnol. 2020. doi:](http://paperpile.com/b/81Zq7k/56jQr)[10.1038/s41587-020-0446-y](http://dx.doi.org/10.1038/s41587-020-0446-y)

9.	[Kim HU, Kim SY, Jeong H, Kim TY, Kim JJ, Choy HE, et al. Integrative genome-scale metabolic analysis of Vibrio vulnificus for drug targeting and discovery. Mol Syst Biol. 2011;7: 460.](http://paperpile.com/b/81Zq7k/7hTE2)

10.	[Haanstra JR, Gerding A, Dolga AM, Sorgdrager FJH, Buist-Homan M, du Toit F, et al. Targeting pathogen metabolism without collateral damage to the host. Sci Rep. 2017;7: 40406.](http://paperpile.com/b/81Zq7k/NHDMp)

11.	[Magnúsdóttir S, Heinken A, Kutt L, Ravcheev DA, Bauer E, Noronha A, et al. Generation of genome-scale metabolic reconstructions for 773 members of the human gut microbiota. Nat Biotechnol. 2017;35: 81–89.](http://paperpile.com/b/81Zq7k/UKMEw)

12.	[Mahadevan R, Schilling CH. The effects of alternate optimal solutions in constraint-based genome-scale metabolic models. Metab Eng. 2003;5: 264–276.](http://paperpile.com/b/81Zq7k/MM2AP)

13.	[Cross GA, Klein RA, Linstead DJ. Utilization of amino acids by Trypanosoma brucei in culture: L-threonine as a precursor for acetate. Parasitology. 1975;71: 311–326.](http://paperpile.com/b/81Zq7k/HHUku)

14.	[Ebrahim A, Lerman JA, Palsson BO, Hyduke DR. COBRApy: COnstraints-Based Reconstruction and Analysis for Python. BMC Syst Biol. 2013;7: 74.](http://paperpile.com/b/81Zq7k/7Xdk)

15.	[Orth JD, Thiele I, Palsson BØ. What is flux balance analysis? Nat Biotechnol. 2010;28: 245–248.](http://paperpile.com/b/81Zq7k/tB6iZ)

16.	[Tielens AG, Van Hellemond JJ. Differences in energy metabolism between trypanosomatidae. Parasitol Today. 1998;14: 265–272.](http://paperpile.com/b/81Zq7k/K2Wxx)

17.	[Sanchez-moreno M. Metabolic studies by 1H NMR of different forms of Trypanosoma cruzi as obtained by “in vitro” culture. FEMS Microbiology Letters. 1995. pp. 119–125. doi:](http://paperpile.com/b/81Zq7k/67S5Q)[10.1016/0378-1097(95)00347-8](http://dx.doi.org/10.1016/0378-1097(95)00347-8)

18.	[Cazzulo JJ, Franke de Cazzulo BM, Engel JC, Cannata JJ. End products and enzyme levels of aerobic glucose fermentation in trypanosomatids. Mol Biochem Parasitol. 1985;16: 329–343.](http://paperpile.com/b/81Zq7k/TyBDo)

19.	[Chavali AK, Whittemore JD, Eddy JA, Williams KT, Papin JA. Systems analysis of metabolism in the pathogenic trypanosomatid Leishmania major. Mol Syst Biol. 2008;4: 177.](http://paperpile.com/b/81Zq7k/4keyf)

20.	[Leroux AE, Maugeri DA, Opperdoes FR, Cazzulo JJ, Nowicki C. Comparative studies on the biochemical properties of the malic enzymes from Trypanosoma cruzi and Trypanosoma brucei. FEMS Microbiol Lett. 2011;314: 25–33.](http://paperpile.com/b/81Zq7k/678uU)

21.	[Lee SH, Stephens JL, Englund PT. A fatty-acid synthesis mechanism specialized for parasitism. Nat Rev Microbiol. 2007;5: 287–297.](http://paperpile.com/b/81Zq7k/QkdN1)

22.	[De Lederkremer RM, Agusti R, Docampo R. Inositolphosphoceramide metabolism in Trypanosoma cruzi as compared with other trypanosomatids. J Eukaryot Microbiol. 2011;58: 79–87.](http://paperpile.com/b/81Zq7k/VkLMt)
