# Supplementary material for: Genome-scale metabolic models highlight stage-specific differences in essential metabolic pathways in Trypanosoma cruzi
Source: PLoS Negl Trop Dis. 2020 Oct 6;14(10):e0008728. doi: 10.1371/journal.pntd.0008728 (PMC7567352; doi:10.1371/journal.pntd.0008728)
Supplement: Attachment — Submitted filename: Rebutal letter_ Aug10.docx [file pntd.0008728.s022.docx]

**Response to the reviewers:**

Reviewer #1: Many thanks to the authors for the answers to the points. The manuscript has greatly approved in the revision.

There are a few remaining points on the revised version. Where relevant, there is a reference to the lines in the Word document that contained the track changes (with the track changes on):

1.	Considering the response to our first point: please indicate in the main text that only 23 genes could be validated (due to limited experimental studies) and that for those genes 79% of the predictions were accurate.

We have made changes in the main text (lines 146-150):

“In addition, we compared the essential reactions predicted by the model iIS312 with the experimental data used in the iSR215 work [13] (See Table S5). iIS312 was able to reproduce 23 out of 29 results from essential gene experiments, which means that 79% of the gene essentiality predictions were accurate (See Table S5).”

2.	Considering the response to our third point: can the authors say something on the MEMOTE results with respect to biomass consistency?

We generated results for all stage-specific models and pan model iIS312 and found that the biomass consistency for pan model iIS312, amastigote model and epimastigote model is 2.19, and the biomass consistency for trypomastigote model is 1.64 (since this biomass reaction was modified). We understand that these values deviate from the ideal value (around 1) for biomass consistency and this is one potential limitation of the model. The biomass reaction of our model was inherited from the *L. major* model (PMID: 18364711) as discussed in the manuscript. This model was built in 2008 and unfortunately lacks molecular weight information, therefore does not conform to our current standard (biomass consistency around 1). Due to the limited data available, we are unable to generate an updated biomass reaction for *T. cruzi* that meet this current standard, but we instead added discussion in the text regarding this limitation, and advise users to be cautious when comparing growth rate of these model with other models (Lines 448-452):

“In addition, regarding the biomass consistency in the MEMOTE report, we found a value of 2.19 for the iIS312, amastigote and epimastigote models, and 1.64 for the trypomastigote model (as the biomass reaction for this stage was modified). Even though the values deviate from the ideal value 1, this result was expected due to limited data for the cellular composition of *T. cruzi*.” For this reason, users should be cautious when comparing the growth rate of iIS312 and stage-specific models with other models.

3.	Considering the response to our fourth point: A very good comparison – we advise to put the most relevant outcomes in the main text

We have made changes in the main text (lines 162-164, and 128-174):

“As indicated in Table S4, out of 2,221 metabolic genes in the transcriptomics data, only 312 were included in our model after checking supportive evidence of their presence in *T. cruzi* metabolism in the literature.”

“In addition, if the gene deactivation resulted in null-growth, gap-filling methods would take place to identify the genes that need to be reactivated to restore growth, and they would be kept active in the downstream analysis (see Methods). Taking the amastigote stage as an example, 68 genes should be deactivated according to the methodology, but only 59 genes (or 42 reactions) were kept deactivated as 9 of them were essential to restore growth to the model. Further discussion about it is presented in the next section.”

4.	It is confusing that at line 135 and Fig 2C the stage-specific models are already used, while the text on how they are made comes only at line 162. Fig 2C could go to Fig 3 and the text about these results too?

Fig 2C became Fig 3B. The passages regarding the end product predictions were moved to the later section.

Because of the order of the text changed, Table S4 became S3, Table S5 became S4, and Table S3 became S5.

5.	The sentence at line 163 is superfluous as lines 165-169 say the same, but are more informative

The passage was rephrased (lines 184-186):

“Therefore, Trypomastigote is the stage with the least active metabolism, followed by amastigote and epimastigote, which is aligned with findings in the literature [4].”

6.	At line 205 there is still a reference to the pan-model. Maybe include the number of the reconstruction as this makes it more clear what the pan model means

All the passages containing “pan model” were rephrased as “pan model iIS312”.

7.	Line 218 suggests that only one reaction is deactivated in epimastigotes, but according to Fig 3 this should be seven reactions.

There was a confusion about which deactivations the paragraph is referring to: this paragraph is introducing a comparison of gene essentiality predictions in the pan model iIS312 and gene deactivations in the stage-specific models. Some changes were made in the passage to make the comparison clearer (lines 211-214, 218-220, and 224-227):

“For amastigotes, the ten of the deactivated genes were found as essential in the pan model iIS312 predictions, and they are enriched in Glycolysis/Gluconeogenesis (see Tables S6 and S7), which is intriguing as the result suggests glycolysis is not essential for the amastigote stage.”

“For trypomastigotes, the deactivated genes that were found as essential in the pan model iIS312 are mostly involved in PPP, which is responsible for nucleic acid synthesis and the production of NADPH.”

“For epimastigotes, the only deactivated gene that was found as essential in the pan model iIS312 is L-threonine dehydrogenase, that is responsible for threonine metabolism, suggesting that threonine may not be essential for parasite growth, or alternative pathways that are missing in the genome annotation are present.”

8.	Line 332/333: a better formulation would be: “…, or enzymes that are more important to sustain glycolytic flux in the pathogen than in the host”.

The passage was rephrased (lines 362-365):

“While these findings reinforced the non-essentiality of glycolysis in amastigotes and suggest the existence of transporters for intermediate glycolytic metabolites from the host cell enzymes that are more important to sustain glycolytic flux in the pathogen than in the host, the essentiality of glycolysis in amastigotes is still being debated as other studies had different observations.”

9.	At line 354-357, please write dpi (days post induction??) in full

The passage was rephrased (lines 358-361):

“2) Li et al. [24,25] showed that glycolytic gene expression levels drastically oscillated over time (0-72 hours post-infection - hpi) - glycolytic gene expression decreased during 0-6hpi when amastigotes infect mammalian cells, and then increased between 6 to 72 hpi, possibly due to the inability of the host to provide intermediate metabolites as it is being invaded by the parasite.”
